# P-1704. Q Fever in Adults: A Comprehensive Overview of Clinical Symptoms and Diagnostic Approaches for Coxiella burnetii in Muscat Oman

**DOI:** 10.1093/ofid/ofaf695.1876

**Published:** 2026-01-11

**Authors:** Hilal Al Sidairi, Surkhab Khan

**Affiliations:** Ministry of Health Oman, Muscat, Masqat, Oman; Oman Medical Specialty Board - Ministry of health Oman, Muscat, Masqat, Oman

## Abstract

**Background:**

Q fever, a globally significant zoonotic disease caused by Coxiella burnetii, poses diagnostic challenges due to nonspecific symptoms. Despite livestock exposure in Oman, human cases remain underreported. This study explores the clinical features of Q fever in febrile patients at the Royal Hospital, highlighting the need for improved awareness, testing, and diagnosis in non-endemic regions.

**Figure:**
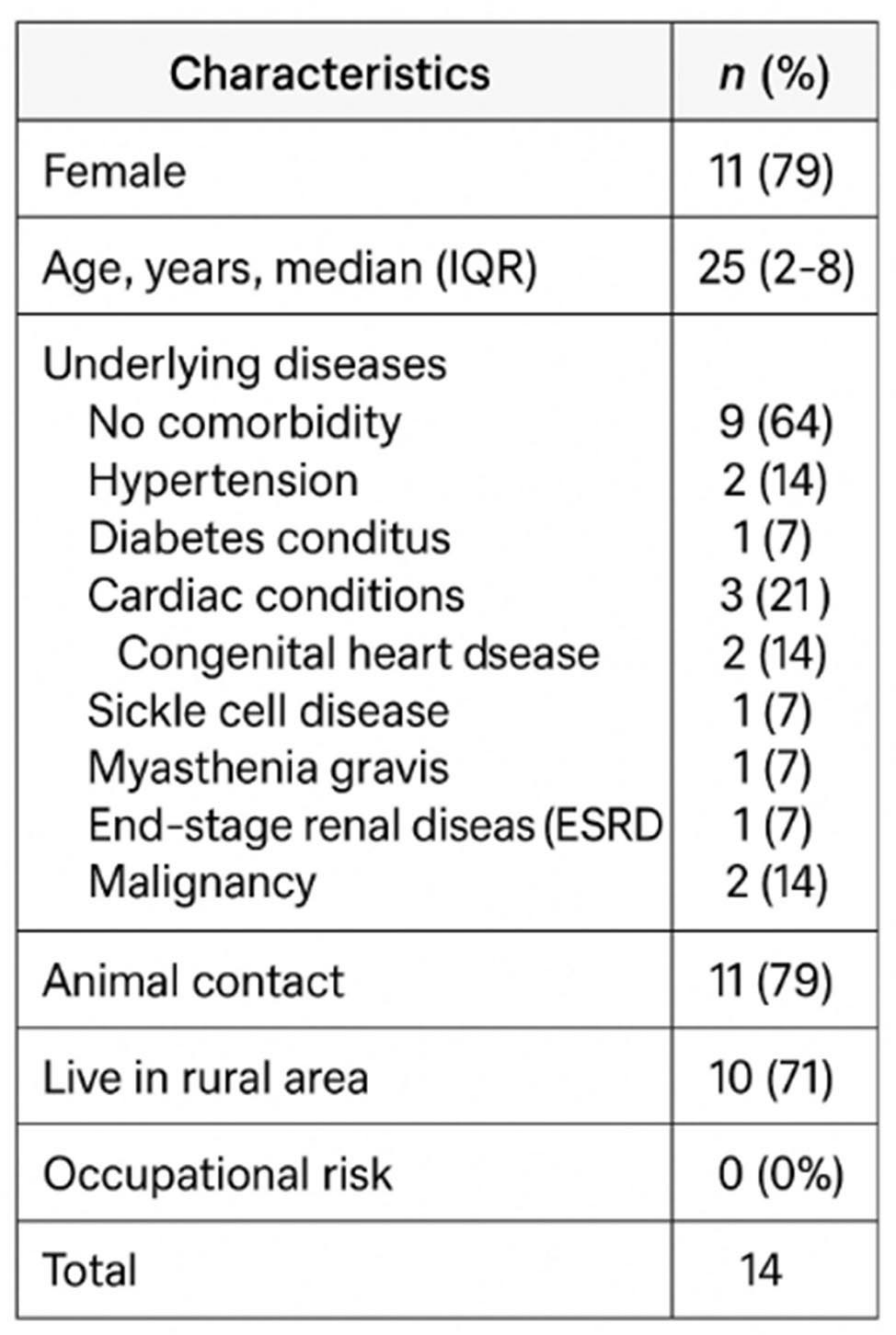
Demographic findings of Q fever patients

**Figure:**
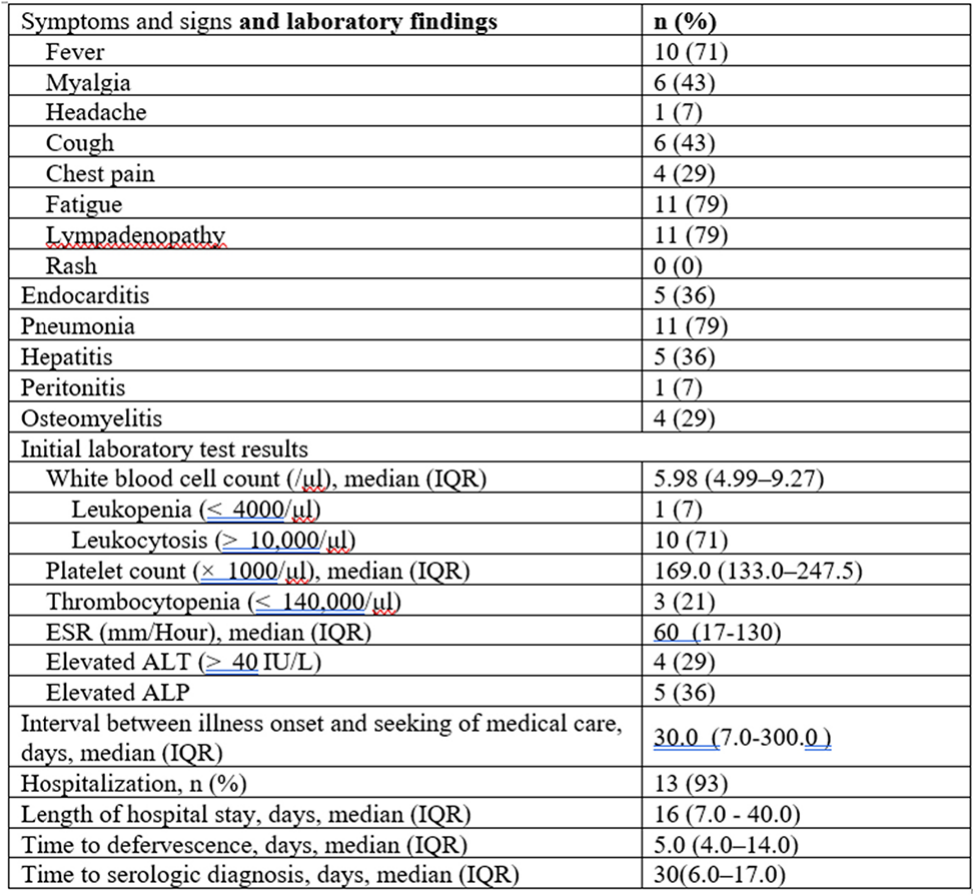
Clinical characteristics and laboratory findings of Chronic Q fever patients (n=14)

**Methods:**

A retrospective descriptive study was conducted at Royal Hospital, Oman (2017–2021), assessing patients tested for Q fever using ELISA, IFA, and PCR. The study collected demographic, clinical, treatment, and outcome data. Cases were classified as acute or chronic based on serologic and molecular criteria. Statistical analysis was performed using SPSS v24, with p < 0.05 considered significant.

**Figure:**
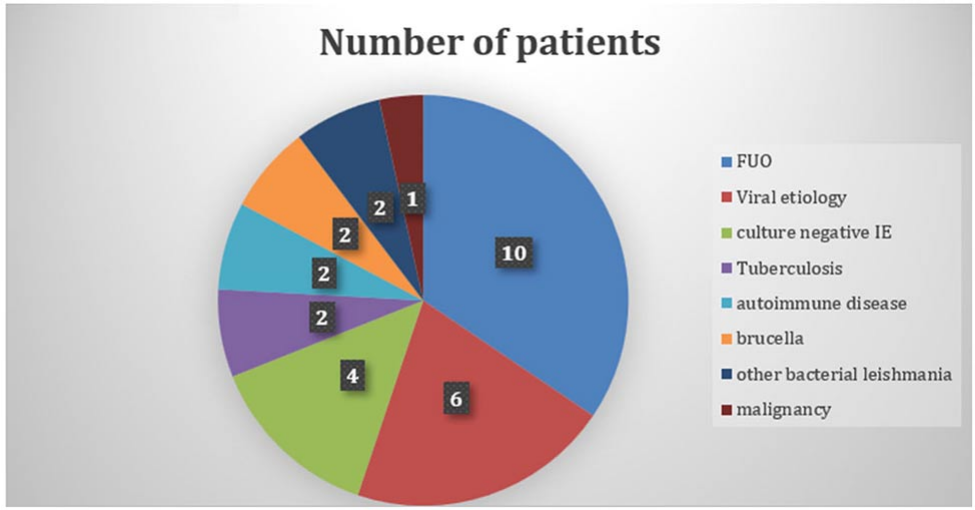
Alternative Diagnoses in ELISA-Positive Patients Not Treated for Q Fever Among 29 patients (15%) who tested ELISA-positive for Q fever but did not receive specific treatment, further investigations revealed alternative diagnoses. Only one patient with infective endocarditis underwent IFA testing, which was negative; no additional confirmatory tests were performed for others. The alternate diagnoses included Leishmania and Brucella infections, autoimmune diseases, viral infections, tuberculosis, pyrexia of unknown origin, and culture-negative cases of endocarditis and osteomyelitis. These findings emphasize the importance of comprehensive differential diagnosis in patients with ELISA-positive results for Q fever.

**Figure:**
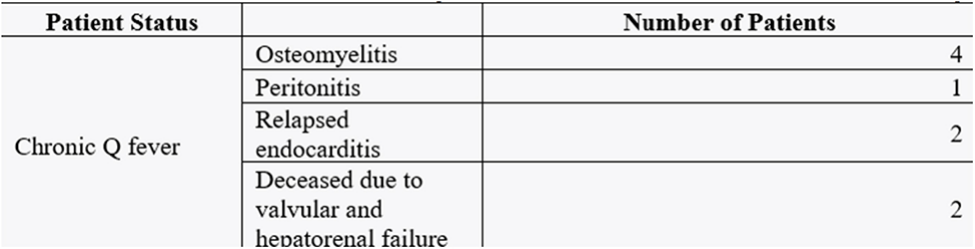
Outcomes and observed complications of the confirmed Q fever patients In our study of 14 confirmed cases (10%), five patients (25%) exhibited clinical features suggestive of probable Q fever, primarily presenting with prolonged fever and nonspecific symptoms like fatigue. Nine patients (50%) progressed to chronic Q fever, developing complications such as chronic joint pain, osteomyelitis, peritonitis, prosthetic joint infections, and recurrent endocarditis. Management required prolonged antibiotic therapy and serial IFA monitoring. Severe outcomes, including valvular heart damage and hepatorenal failure, occurred in two patients (10%). These findings highlight the diverse manifestations and significant clinical burden of chronic Q fever

**Results:**

Twenty confirmed cases of Q fever were identified, predominantly among young females from rural areas with documented animal exposure. Clinical presentations ranged from nonspecific fever to more severe manifestations such as pneumonia and endocarditis. Diagnosis was primarily achieved through ELISA, with a median time to confirmation of 32 days. Fourteen patients progressed to chronic Q fever, often requiring prolonged treatment due to complications. These findings underscore the importance of clinical awareness and timely diagnosis of Q fever, particularly in non-endemic regions where it may be easily overlooked.

**Conclusion:**

This study emphasizes the under-recognition of Q fever in Oman, urging increased awareness among healthcare professionals, particularly in non-endemic areas. The clinical presentation is diverse, with pneumonia being the most common manifestation, and chronic Q fever linked to complications like endocarditis. Despite Q fever's endemic status, there is a lack of comprehensive epidemiological data and surveillance. Delays in diagnosis were noted due to the disease’s varied clinical presentations. Additionally, the study found a higher prevalence of Q fever in females, contrary to global trends, suggesting region-specific factors. A multifaceted diagnostic approach, including serologic testing, PCR, and clinical evaluation, is vital for accurate diagnosis and effective management.

**Disclosures:**

All Authors: No reported disclosures

